# Phytotoxicity of Zero-Valent Iron-Based Nanomaterials in Mung Beans: Seed Germination and Seedling Growth Experiments

**DOI:** 10.3390/toxics13040250

**Published:** 2025-03-27

**Authors:** Huan Wu, Sha Li, Yu He, Bin Zhou, Guoming Zeng, Yuanyuan Huang, Da Sun

**Affiliations:** 1 Intelligent Construction Technology Application Service Center, Chongqing City Vocational College, Chongqing 402160, China; color.wu@163.com (H.W.); 15111854724@163.com (S.L.); 15823030885@163.com (B.Z.); 2017015@cqust.edu.cn (G.Z.); 2 National & Local Joint Engineering Research Center for Ecological Treatment Technology of Urban Water Pollution, College of Life and Environmental Science, Wenzhou University, Wenzhou 325035, China; i136897502@163.com; 3 Chongqing Academy of Science and Technology, Chongqing 401123, China; 4 School of Civil Engineering, Chongqing Jiaotong University, Chongging 400074, China; 5 Zhejiang Provincial Key Laboratory for Water Environment and Marine Biological Resources Protection, College of Life and Environmental Science, Wenzhou University, Wenzhou 325035, China; 6 Institute of Life Sciences, Biomedical Collaborative Innovation Center of Zhejiang Province, Wenzhou University, Wenzhou 325035, China

**Keywords:** nano-zero-valent iron, mung bean, biochar loading modification, toxicity, plant vigor

## Abstract

The extensive utilization of nano-zero-valent iron (nZVI) and its engineered derivatives has prompted significant environmental concerns, particularly regarding their phytotoxicological impacts, which remain inadequately characterized. This investigation systematically evaluated the phytotoxicological responses induced by nZVI, Chlorella vulgaris biochar (BC), and Chlorella vulgaris biochar loaded with nano-zero-valent iron (BC/nZVI) on mung bean seed germination and subsequent seedling development. The experimental data revealed that both the nZVI and BC/nZVI treatments significantly suppressed the germination indices, including germination rate, radicle and plumule elongation, and biomass accumulation, with nZVI demonstrating the most pronounced inhibitory effects. During the vegetative growth phases, nZVI exposure substantially impaired plant morphogenesis, manifested through reduced vertical growth, diminished fresh and dry biomass production, and the onset of premature foliar chlorosis, necrosis, desiccation, and, ultimately, plant mortality. A comparative analysis indicated that the BC/nZVI composites exhibited less severe photosynthetic inhibition relative to pristine nZVI. Biochemical assays demonstrated that nZVI exposure elicited the substantial upregulation in antioxidant enzyme activities, including superoxide dismutase (SOD), catalase (CAT), and peroxidase (POD), concomitant with abnormal ferric ion accumulation in root tissues. Notably, BC/nZVI composites demonstrated the partial mitigation of these physiological disturbances. These empirical findings underscore that excessive iron bioavailability from nZVI induces substantial phytotoxicological stress, while BC matrix incorporation provides the partial amelioration of these adverse effects on seedling ontogeny.

## 1. Introduction

Nano-zero-valent iron (nZVI) is a promising material for environmental remediation. However, while effectively targeting pollutants, nZVI can also disrupt ecosystems and adversely affect the organisms within them [1]. In recent years, the extensive use of nZVI has raised concerns regarding its environmental risks, particularly its ecotoxicity to plants, microorganisms, and animals [2,3].

Iron, as one of the essential trace elements for plant growth, plays a crucial role in many physiological processes. It is an important component in the synthesis of chlorophyll, contributing to photosynthesis and the overall vitality of plants. Iron also serves as a cofactor for enzymes involved in the electron transport chain, such as cytochromes and iron–sulfur proteins. They are vital for energy production, redox reactions, and the synthesis of DNA and hormones [4,5]. However, research has shown that nZVI can also exert significant toxic and excitatory effects on plant growth and development [6,7]. For example, Brasili et al. used nano-zero-valent iron (nZVI) as a seed initiator to investigate its effect on tomato seed germination and seedling growth. The results showed that low concentrations of nZVI (5 mg/L and 50 mg/L) positively impacted seed germination and significantly increased seedling biomass and photosynthetic pigment content. However, higher concentrations (100 mg/L and 1000 mg/L) had a significant negative effect on seed germination [8]. This effect is not universal; Li et al. found that a low concentration of nZVI (17.92 mg/L) significantly inhibited peanut plant growth [9]. Conversely, Kim et al. exposed *Arabidopsis thaliana* to a high concentration of nZVI (500 mg/L) and observed a significant increase in root elongation [10].

The phytotoxicological responses to nano-zero-valent iron (nZVI) exhibit significant interspecies variability and are substantially influenced by surface functionalization strategies, as evidenced by previous studies [11,12,13]. A notable investigation by Ma et al. demonstrated differential cellular uptake patterns, wherein nZVI particles successfully penetrated root cell membranes in Populus spp. but were effectively excluded in Psoralea corylifolia, primarily attributable to the latter’s enhanced lignification of cell walls serving as an effective physicochemical barrier against exogenous nanomaterials [14]. Under environmental exposure conditions, nZVI undergoes rapid oxidative transformation to ferrous (Fe^2+^) and ferric (Fe^3+^) species, accompanied by substantial particle agglomeration phenomena [15,16]. To address these limitations, surface modification methodologies have been extensively employed to optimize nZVI’s colloidal stability and redox reactivity, with substantial empirical evidence supporting the efficacy of engineered nZVI composites in environmental remediation applications targeting contaminated matrices [17,18,19]. Nevertheless, the ecotoxicological implications of surface modifications remain controversial. While some investigations propose that optimized surface coatings can attenuate nZVI’s bioadhesion through synergistic electrostatic repulsion and steric hindrance mechanisms, thereby reducing its biological impacts [20], the overall toxicological profile remains complex and context-dependent. Conversely, Yoon et al. found that both carboxymethyl cellulose (CMC)-stabilized nZVI and bismuth-doped nZVI exhibited strong toxicity toward *Arabidopsis thaliana*, with the toxicity ranking as follows: Bi-CMC > CMC-nZVI > bare nZVI [21]. On the other hand, Zhou et al. reported that CMC acts as a free radical scavenger, reducing nZVI’s toxicity by competing with bacteria for oxidants [22]. Furthermore, Kadar et al. observed that modified nZVI increased sperm toxicity in purple mussel embryos compared to bare nZVI, likely due to the surface coating enhancing nZVI stability during organism contact [23]. These findings indicate that the effects of surface modification on nZVI phytotoxicity are unclear, warranting further investigation.

Despite the widespread use of biochar-loaded nZVI for environmental remediation, the phytotoxicity of load-modified nZVI has rarely been reported [24,25,26,27,28]. Therefore, this study aimed to investigate the biological effects of a biochar-loaded nZVI composite on plants. Chlorella powder was selected as a stabilizer because of its cost-effectiveness and potential for resource utilization of algae species. In previous research, Chlorella vulgaris biochar loaded with nano-zero-valent iron (BC/nZVI) was used to remove *Microcystis aeruginosa* from water bodies [29]. Mung beans have been widely used in phytotoxicity studies due to their well-documented responses to environmental stressors, providing a reliable basis for comparison with other research findings. To further assess its ecological safety, this study systematically analyzed the effects of Chlorella vulgaris biochar (BC), nZVI, and BC/nZVI on mung beans through seed germination and seedling growth tests. First, the impact of these treatments on the germination rate, root and shoot length, and dry and fresh weights of the mung bean seeds was evaluated to determine their effects on the germination process. Next, the seedling growth test investigated the influence of the treatments on the dry and fresh weights, stem and leaf root lengths, and photosynthetic pigment content of mung bean seedlings, exploring the mechanisms of their impact on mung bean growth and physiological and biochemical indexes. Additionally, the study examined the oxidative metabolic damage in mung beans by analyzing changes in antioxidant enzyme activities before and after the treatments.

## 2. Materials and Methods

### 2.1. Seed Germination Test

Mung bean (*Vigna radiata*) seed is a general-purpose agricultural seed purchased from the local market in Chongqing, China. Seeds of uniform size and full grain were randomly selected and soaked in 75% alcohol (ChengDu Chron Chemicals Co., Ltd., Chengdu, China) for 2 min for surface disinfection. After disinfection, they were rinsed with deionized water 5 times and then dried with filter paper. Then, the sterile solutions of Chlorella biochar (BC), nano-ferric zero-valent (nZVI), and nano-ferric zero-valent (BC/nZVI)-loaded Chlorella biochar were prepared with deionized water at a concentration of 12.5 g L^−1^. The Supplementary Documents provide the preparation methods for the BC, nZVI, and BC/nZVI materials. For more detailed preparation processes and material properties, please refer to our previous research [30]. Then, three petri dishes were added with 15 mL of prepared BC, nZVI, and BC/nZVI solution, respectively, as the experimental group. Another petri dish was added with 15 mL deionized water as a blank control. Ten mung bean seeds were placed in each petri dish, ensuring that the seed spacing is greater than 1 cm. All petri dishes were placed in an artificial climate incubator (Shanghai Yiheng Scientific Instrument Co., Ltd., Shanghai, China) for germination tests in dark conditions of 25 °C and 70% relative humidity. Three parallel samples were set up for each treatment.

#### 2.1.1. Determination of Seed Germination

When the length of the germination exceeds half of the seed length, the germination is considered complete. After 5 days of culture, the number of germinated seeds was recorded, and the germination rate was calculated using the following formula:

(1) $$\mathrm{germination}\;\mathrm{rates}\left(\%\right)=\frac{\mathrm{Number}\;\mathrm{of}\;\mathrm{seeds}\;\mathrm{germinating}\;\mathrm{normally}}{\mathrm{Number}\;\mathrm{of}\;\mathrm{seeds}\;\mathrm{for}\;\mathrm{testing}}\times 100\%$$

#### 2.1.2. Determination of Fresh Dry Weight of Seeds

After harvest, the length of the seed root buds was measured using a millimeter scale. The root buds were thoroughly rinsed with deionized water and dried to remove the surface moisture. The fresh weight (FW) of the seeds was then measured using an electronic balance and recorded. After weighing, the seeds were heat-treated in an oven at 100–105 °C for 10 min. The temperature was then reduced to 70–80 °C, and the drying continued until a constant weight was achieved. Finally, the dry weight (DW) of the seeds was recorded after cooling the samples to room temperature in a desiccator.

### 2.2. Seedling Growth Test

Seeds are selected and sterilized, as described in the germination test. They were then seeded in colonial baskets, with 10 seeds evenly distributed in each basket and filled with sterilized perlite (a diameter of 2 mm). The basket is placed in an artificially controlled plant growth chamber with a day/night cycle of 16/8 h, with a daytime temperature of 28–33 °C and a night temperature of 20–25 °C, light intensity of 10,000 Lux, and relative humidity of about 70%. On day 20 after seeding, the Hoagland nutrient solution (Qingdao Haibo Biological Co., Ltd., Qingdao, China) was added to the BC, nZVI, or BC/nZVI solutions at 12.5 g L^−1^ for 3 replicates. Finally, the seedlings are harvested after the third day.

#### 2.2.1. Measurement of Growth Indicators

Prior to harvest, the aboveground height of the plants was measured using a millimeter scale and recorded. At harvest, the plants were divided into aboveground and belowground parts. The aboveground parts were washed with deionized water to remove the surface dust and then dried to determine their fresh weight. The root systems were soaked in a 10% sodium ethylenediaminetetraacetic acid (EDTA, Shanghai Maclin Biotechnology Co., Ltd., Shanghai, China) buffer for 20 min, rinsed again, and wiped dry before measuring their fresh weight. The fresh plant samples were then killed at 105 °C for 10 min, followed by drying in an oven at 70 °C for 24 h. Finally, the dry weights of both aboveground and belowground parts were measured using a precision electronic balance with a sensitivity of 1 in 10,000.

#### 2.2.2. Measurement of Photosynthetic Pigment Content

Before harvesting the mung bean seedlings, the second true leaf from the top was excised for phytochlorophyll content determination. After removing the main leaf veins, the leaf was cut into small pieces, weighed to 0.1 ± 0.0001 g, and placed in 25 mL of 95% ethanol (ChengDu Chron Chemicals Co., Ltd., Chengdu, China). The leaf pieces were soaked overnight until they turned white, indicating the completion of the leaching process. The absorbance of chlorophyll a, chlorophyll b, and carotenoids was measured at 649 nm, 665 nm, and 470 nm, respectively, using a UV–visible spectrophotometer (DR6000, HACH, Loveland, CO, USA). Each measurement was performed in triplicate, and the mean values along with the standard errors (SEs) were calculated to assess the variability. The standard errors were derived from the standard deviation (SD) of the replicates divided by the square root of the sample size (*n*). The concentrations of these pigments were calculated using the following equation [31]:

(2) $$C_{a}=13.95A_{665}-6.88A_{649}$$

(3) $$C_{b}=24.65A_{649}-7.32A_{665}$$

(4) $$C_{d}=\frac{1000A_{470}-2.05C_{a}-114.8C_{b}}{245}$$

where C_a_, C_b_, and C_d_ are chlorophyll a, chlorophyll b, and carotenoids, respectively. A_665_, A_649_, and A_470_ represent the absorbance values at 665, 649, and 470 nm, respectively.

#### 2.2.3. Antioxidant Enzyme Activity Assay

Accurately weigh 0.1 g of plant tissue from both aboveground and belowground parts. Add four times the weight of the homogenization medium (0.1 mol/L PBS, pH 7.0) to the samples, maintaining a weight-to-volume ratio of 1:4. Prepare a 20% homogenate using a mechanical homogenizer while maintaining the samples in an ice water bath. Centrifuge the homogenate at 4000 rpm for 10 min and then collect the supernatant for analysis. Subsequently, measure the activities of superoxide dismutase (SOD), peroxidase (POD), and catalase (CAT) using assay kits (purchased from Nanjing Jiancheng Bioengineering Institute, China) according to the manufacturer’s instructions.

#### 2.2.4. Determination of Elemental Fe Content

The dried plant samples were ground to a particle size of less than 0.25 mm. A 0.5 g aliquot of both aboveground and belowground dry samples was digested using high-purity nitric acid (HNO_3_) under controlled conditions to ensure complete dissolution of the samples. The resulting digest was diluted to an appropriate volume with deionized water and filtered to remove any particulate matter. The Fe concentration in the digest was determined using inductively coupled plasma optical emission spectrometry (ICP-OES; iCAP 7200, Thermo Fisher Scientific, Waltham, MA, USA). The instrument was operated under standard conditions, and all measurements were performed in triplicate to ensure reproducibility. The results are reported as mean values with standard deviations.

#### 2.2.5. TEM Analysis

The roots of the plant seedlings were removed and placed in a 2.5% glutaraldehyde solution (prepared in PBS) for fixation overnight at 4 °C. After the fixation, the samples were washed four times with PBS and then fixed in a 1% osmium tetroxide solution overnight at 4 °C, followed by four additional PBS washes. The samples were then dehydrated with a graded ethanol series, replaced with acetone, and embedded in a curing agent for over 48 h. Subsequently, sections approximately 2–3 cm above the root tip were cut using a Leica EM UC7 ultramicrotome (Leica Microsystems, Wetzlar, Germany) and examined under a transmission electron microscope (TEM) (Tecnai G2 Spirit, FEI Corporation, Hillsboro, OR, USA).

### 2.3. Data Processing

Experimental data were processed using Origin 2022 (Version 9.7) and the IBM SPSS Statistics (Version 19) software. To compare the significance between the different treatments, Duncan’s multipole difference test (*p* < 0.05) and a one-way analysis of variance were used.

## 3. Results and Discussion

### 3.1. Seed Germination Experiment

The effects of the BC, nZVI, and BC/nZVI composite on mung bean seed germination were examined at a concentration of 12.5 g·L^−1^ (see Supplemental Materials). BC demonstrated superior dispersion characteristics in aqueous media, correlating with enhanced seed germination performance. Comparative analysis revealed distinct morphological responses among the treatment groups: nZVI-exposed seeds displayed significant radicle growth inhibition and compromised germination efficiency, while BC/nZVI-treated specimens exhibited successful radicle emergence through the test with subsequent development of primary root systems. A quantitative assessment of the germination parameters showed relative inhibition rates of 18.97%, 41.38%, and 24.14% for the BC, nZVI, and BC/nZVI treatments, respectively, compared to the control conditions. A statistical analysis (*p* < 0.05) confirmed that nZVI treatment significantly suppressed the seed development parameters relative to both the control and BC-containing experimental groups, establishing a clear phytotoxicological hierarchy among the treatments.

BC, nZVI, and BC/nZVI also affected the root and shoot lengths (see Supplemental Materials). nZVI had a significantly greater impact on shoot length, reducing it by 86.10% compared to the control, more so than BC and BC/nZVI (*p* < 0.05). Similarly, nZVI reduced the root length by 95.10%, significantly more than BC and BC/nZVI (*p* < 0.05).

At a 12.5 g·L^−1^ concentration, BC, nZVI, and BC/nZVI all influenced plant fresh weight compared to the control (see Supplemental Materials). Significant differences (*p* < 0.05) were observed, with reductions of 25.25% in the BC group, 42.40% in the nZVI group, and 31.75% in the BC/nZVI group. The nZVI group had the most substantial effect on fresh weight. However, the effects on dry weight were minimal and not significantly different among the groups (*p* > 0.05). This indicates that the BC/nZVI composite had a significantly lower toxic effect on mung bean seeds compared to nZVI alone.

### 3.2. Seedling Growth Experiment

#### 3.2.1. Effect on Growth Index of Mung Bean Seedlings

**Figure 1:**
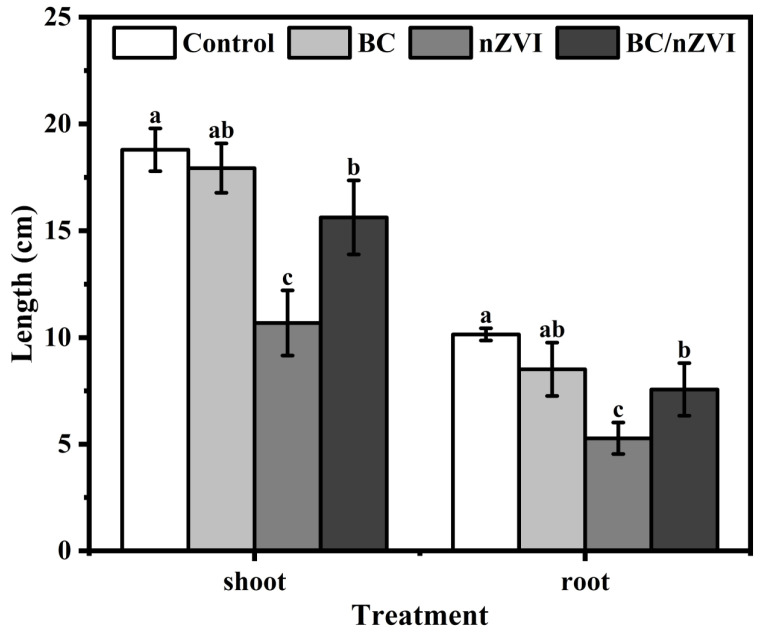
Plant height and root length of mung bean seedlings after 72 h exposure to BC, nZVI, and BC/nZVI. Lower letters indicate significant differences in mean values within BC, nZVI, and BC/nZVI treatment groups (*p* < 0.05). BC: Chlorella vulgaris biochar; nZVI: nano-zero-valent iron; BC/nZVI: Chlorella vulgaris biochar loaded with nano-zero-valent iron.

For plant height (Figure 1), BC did not significantly affect mung bean seedling height compared to the control group (*p* > 0.05). However, nZVI treatment led to a substantial reduction in plant height, decreasing it by 43.13% compared to the control group. The nZVI group showed significantly lower seedling height compared to both the control and BC/nZVI groups (*p* < 0.05), suggesting a stronger inhibitory effect of nZVI on growth. While the BC/nZVI composite treatment demonstrated statistically significant (*p* < 0.05) inhibition of vertical plant growth relative to the control conditions, its phytotoxic effect was markedly attenuated compared to pristine nZVI exposure (*p* < 0.05). This growth inhibition pattern was consistently observed in root system development, with treatment effects exhibiting parallel trends to those recorded for shoot morphogenesis. A quantitative analysis revealed differential root length reduction percentages of 16.10%, 48.01%, and 25.42% for BC, nZVI, and BC/nZVI treatments, respectively, when compared to the untreated controls. The consistent response patterns between aerial and subterranean growth parameters suggest a systemic phytotoxic effect mediated through similar physiological mechanisms.

#### 3.2.2. Effects on Mung Bean Seedling Biomass

**Figure 2:**
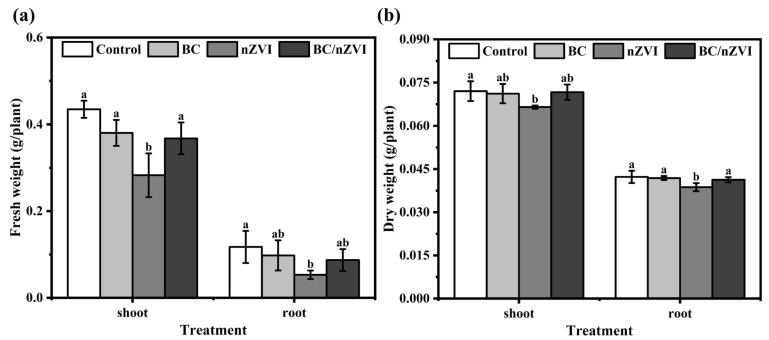
Fresh weight (**a**) and dry weight (**b**) of mung bean seedlings after 72 h of exposure to BC, nZVI, and BC/nZVI. Lower letters indicate significant differences in mean values within BC, nZVI, and BC/nZVI treatment groups (*p* < 0.05). BC: Chlorella vulgaris biochar; nZVI: nano-zero-valent iron; BC/nZVI: Chlorella vulgaris biochar loaded with nano-zero-valent iron.

The effects of BC, nZVI, and BC/nZVI at a concentration of 12.5 g L^−1^ on the dry and fresh weights of plants varied (Figure 2). A comparative biomass analysis revealed statistically significant (*p* < 0.05) differences in both fresh and dry biomass accumulation in the aerial and root tissues of Vigna radiata seedlings between nZVI-treated specimens and those exposed to BC or BC/nZVI composites. Notably, no significant biomass variations (*p* > 0.05) were detected when comparing BC/nZVI-treated plants with either the control or BC-only groups. These findings demonstrate that BC incorporation effectively attenuates the phytotoxic effects of nZVI, as evidenced by the restoration of normal biomass accumulation patterns in BC/nZVI-treated seedlings compared to those exposed to unmodified nZVI.

#### 3.2.3. Photosynthetic Pigment Content

**Figure 3:**
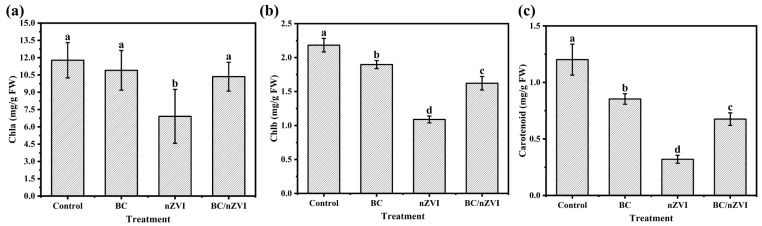
Chlorophyll a (**a**), chlorophyll b (**b**), and carotenoid content (**c**) in the leaves of mung bean seedlings. Lower letters indicate significant differences in mean values within the BC, nZVI, and BC/nZVI treatment groups (*p* < 0.05). BC: Chlorella vulgaris biochar; nZVI: nano-zero-valent iron; BC/nZVI: Chlorella vulgaris biochar loaded with nano-zero-valent iron.

Iron (Fe) is the most absorbed trace element in plants [32] and plays a crucial role in chlorophyll synthesis, which is essential for maintaining the structure and function of chloroplasts [33,34]. In this study, all three treatments—BC, nZVI, and BC/nZVI—negatively affected chlorophyll a, chlorophyll b, and carotenoid content in mung bean leaves (Figure 3). The nZVI treatment had the most substantial impact, reducing chlorophyll a, chlorophyll b, and carotenoid content by 41.32%, 50.10%, and 73.33%, respectively, compared to the control. A spectrophotometric analysis of the photosynthetic pigments revealed differential inhibition patterns among the treatment groups. The BC/nZVI exposure resulted in significant (*p* < 0.05) reductions of 12.08%, 25.68%, and 43.78% in chlorophyll a, chlorophyll b, and carotenoid concentrations, respectively. Comparatively, the BC treatment demonstrated less pronounced pigment degradation, with corresponding decreases of 7.42%, 13.08%, and 28.97% for the same photosynthetic components. A statistical analysis confirmed the significant inter-group differences (*p* < 0.05) in the pigment inhibition rates between the BC and BC/nZVI treatments, suggesting that nZVI incorporation into the BC matrix exacerbates photosynthetic pigment degradation despite the protective effects of biochar.

#### 3.2.4. Antioxidant Enzyme System Activity

Under external stress, plants stabilize cell membranes by scavenging excess reactive oxygen species (ROS) and inhibiting lipid peroxidation through antioxidant enzymes, such as superoxide dismutase (SOD), catalase (CAT), and peroxidase (POD) [35,36].

**Figure 4:**
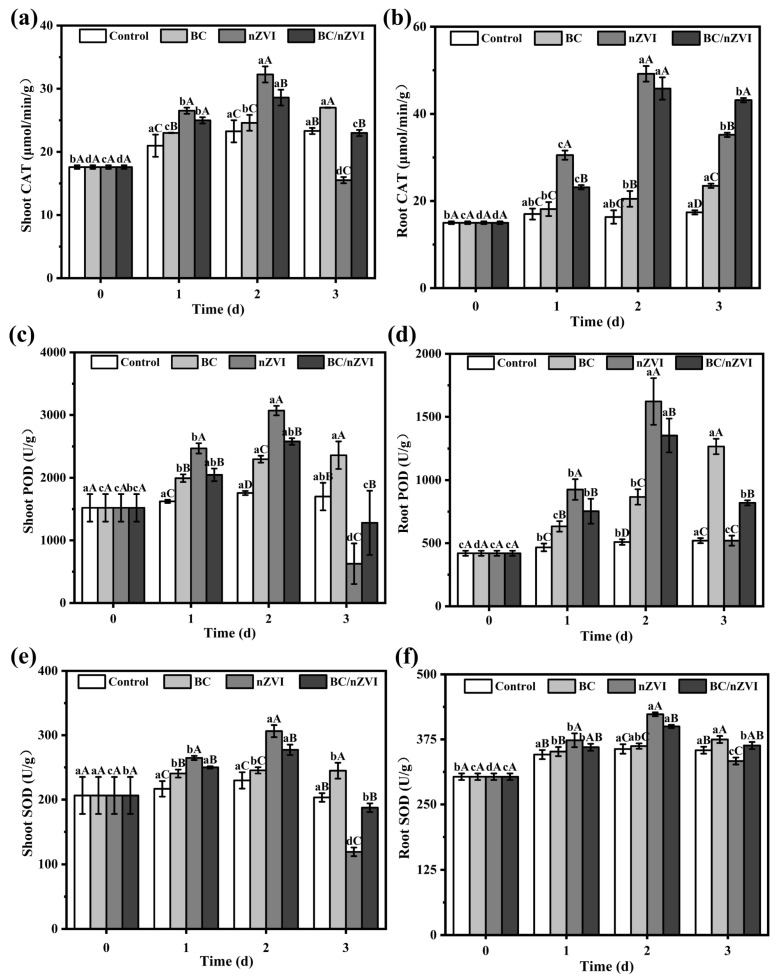
Effects of BC, nZVI, and BC/nZVI on CAT, POD, and SOD enzyme activities in mung bean seedlings. (**a**,**c**,**e**) are the aboveground portion; (**b**,**d**,**f**) are the underground portion. Lower- and uppercase letters indicate significant differences in the mean values within and between the BC, nZVI, and BC/nZVI treatment groups, respectively (*p* < 0.05). BC: Chlorella vulgaris biochar; nZVI: nano-zero-valent iron; BC/nZVI: Chlorella vulgaris biochar loaded with nano-zero-valent iron.

SOD converts superoxide anion (·O^2−^) into hydrogen peroxide (H_2_O_2_), while CAT and POD further break down H_2_O_2_ into water, reducing its cellular toxicity. As shown in Figure 4, the SOD, CAT, and POD levels in mung bean leaves and roots treated with biochar (BC) exhibit a gradual increase compared to the control group. In contrast, the nZVI and BC/nZVI groups displayed an initial increase followed by a decrease. On the third day, the enzyme level of the BC treatment group was stable, and the contents of SOD, CAT, and POD reached 244.90 U/g, 27.00 μmol/min/g, and 2360.00 U/g, respectively, whereas those in the nZVI and BC/nZVI groups declined significantly, becoming notably lower than those in the BC group (*p* < 0.05). The nZVI group exhibited the most pronounced fluctuations, with the enzyme activity in the mung bean leaves falling below that of the control group. This indicates that BC, nZVI, and BC/nZVI exert different oxidative stress effects on mung bean plants. Mung bean seedlings exposed to 12.5 g·L^−1^ of BC showed minimal oxidative stress, whereas those treated with nZVI and BC/nZVI activated protective responses against stress but also exhibited some inhibitory effects, with nZVI showing the highest toxicity.

#### 3.2.5. Effect of BC/nZVI on Iron Content of Mung Bean Seedlings

**Figure 5:**
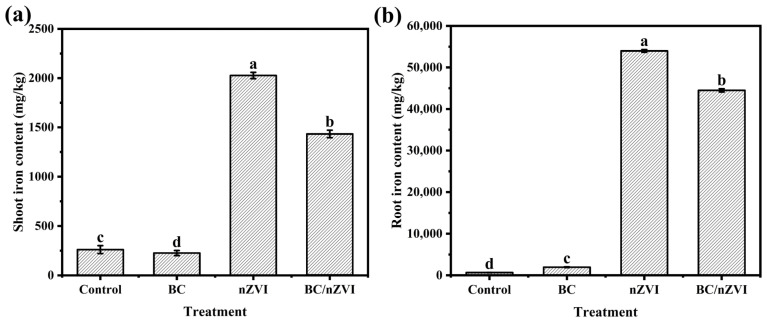
Effects of BC, nZVI, and BC/nZVI on iron content of mung bean seedlings. (**a**) Aboveground portion. (**b**) Underground portion. Lower letters indicate significant differences in the mean values within the BC, nZVI, and BC/nZVI treatment groups (*p* < 0.05). BC: Chlorella vulgaris biochar; nZVI: nano-zero-valent iron; BC/nZVI: Chlorella vulgaris biochar loaded with nano-zero-valent iron.

The data presented in Figure 5 demonstrate that both the nZVI and BC/nZVI treatments enhance iron uptake in the root system of mung bean plants. An elemental analysis revealed distinct iron translocation patterns among the treatment groups, with nZVI exposure demonstrating the most pronounced iron accumulation in mung bean seedlings. A quantitative assessment showed that nZVI-treated plants exhibited 7.76-fold and 81.40-fold increases in iron concentration in aerial tissues and root systems, respectively, compared to the control specimens. Similarly, the BC/nZVI treatment significantly enhanced iron bioavailability, resulting in 5.49-fold and 67.08-fold elevations in iron content in aboveground and belowground tissues, respectively, relative to the untreated controls. These findings demonstrate that while BC modification attenuates iron uptake, BC/nZVI composites still substantially influence iron translocation dynamics in mung bean seedlings, suggesting that biochar modification only partially mitigates the enhanced iron bioavailability associated with nZVI exposure.

#### 3.2.6. TEM Analysis

**Figure 6:**
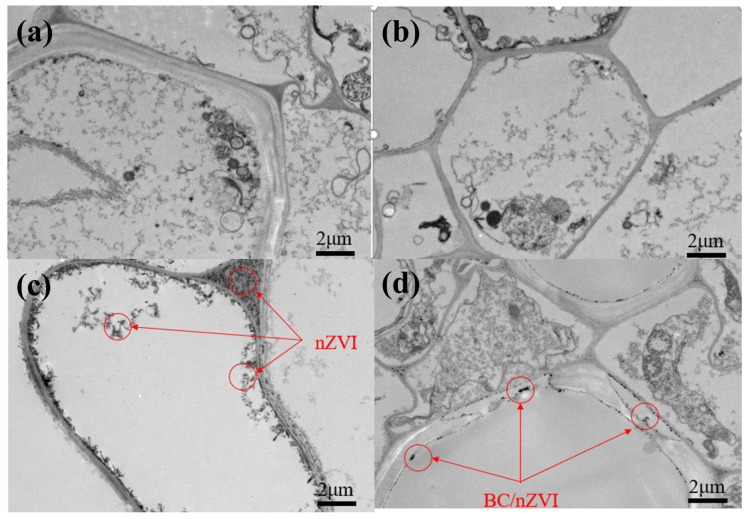
TEM images of the roots of mung bean seedlings in the control group and after exposure to different material treatments. (**a**) Control group. (**b**) BC experimental group. (**c**) nZVI experimental group. (**d**) BC/nZVI experimental group. BC: Chlorella vulgaris biochar; nZVI: nano-zero-valent iron; BC/nZVI: Chlorella vulgaris biochar loaded with nano-zero-valent iron.

High concentrations of Fe were detected in mung bean roots exposed to nZVI and BC/nZVI, prompting TEM observations to assess changes in the root cellular structure. As shown in Figure 6, the control and BC-treated mung bean roots display normal cellular and subcellular structures. A cytological analysis revealed distinct ultrastructural alterations among the treatment groups. The nZVI-exposed root cells displayed pronounced plasmolysis phenomena and cytoplasmic condensation, accompanied by extensive deposition of electron-dense aggregates at multiple subcellular locations, including the cell wall interface, plasma membrane periphery, and intracellular compartments. In contrast, the BC/nZVI-treated specimens maintained relatively preserved cellular architecture, with only sporadic extracellular deposition of particulate matter. These ultrastructural observations provide compelling evidence that biochar functionalization substantially attenuates the cytological impacts of nZVI, suggesting a protective mechanism through reduced cellular internalization and membrane interaction of nZVI particles.

### 3.3. Analysis of Virulence Mechanisms

The application of iron-based restorative materials increased the iron content in mung beans to some extent. Two distinct strategies have evolved in higher plants for Fe uptake [37]. Plant roots typically enhance their reducing capacity for Fe^3+^ by releasing Fe^3+^-chelate reductase, which promotes the biosynthesis of Fe^2+^ transporter proteins and inter-root acidification [38]. In legumes, iron uptake follows a reduction-based mechanism (Strategy I), where root excretion of protons reduces Fe^3+^ to labile Fe^2+^, which is then transported into root cells via the Fe^2+^ transporter protein IRT1. Consequently, applying high concentrations of nZVI and BC/nZVI led to an increase in Fe content in mung bean roots.

**Figure 7:**
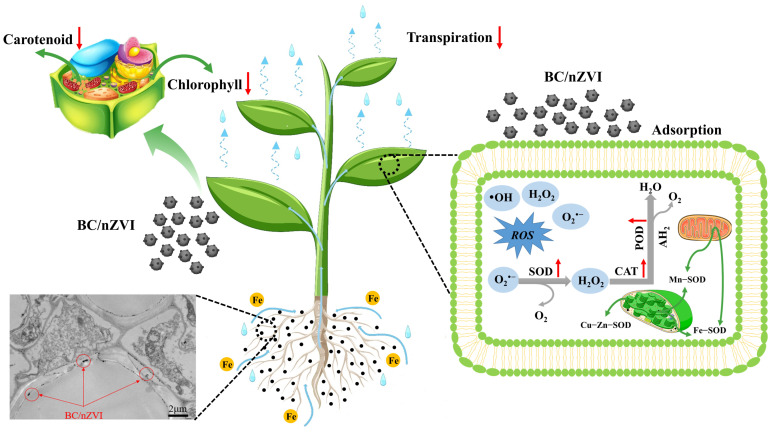
Mechanisms of BC/nZVI toxicity. BC: Chlorella vulgaris biochar; nZVI: nano-zero-valent iron; BC/nZVI: Chlorella vulgaris biochar loaded with nano-zero-valent iron.

Iron is crucial for chlorophyll synthesis and chloroplast function [33,39]. Iron deficiency in plants can cause significant alterations to the lamellar structure of chloroplasts and, in severe cases, lead to chloroplast disintegration. In this study, we measured the photosynthetic pigment content in plant leaves to evaluate the effects of nZVI and BC/nZVI on chloroplasts and photosynthesis. Overall, the application of nZVI and BC/nZVI inhibited the synthesis of chlorophyll a, chlorophyll b, and carotenoids in mung bean leaves. The excessive transfer of Fe ions to the stems and leaves of the nZVI- and BC/nZVI-treated plants likely disrupted chloroplast function, preventing the utilization of the accumulated Fe. This disruption led to leaf yellowing, chlorotic veins, and reduced growth rates [40]. The induction of ROS production, leading to oxidative damage, is a well-known mechanism of nZVI toxicity [41,42]. Under excess iron conditions, Fe^2+^ catalyzes the formation of highly toxic ROS through the Fenton reaction [43]. The accumulation of ROS induces oxidative stress in plants, triggering the activation of antioxidant defenses, including glutathione, ascorbate, and antioxidant enzymes [44,45]. In this study, the nZVI and BC/nZVI treatments led to significant increases in the activities of peroxidase enzymes (SODase, CATase, and PODase), indicating that excess iron stimulated the production of H_2_O_2_ and ·O^2−^, causing cellular toxicity. However, as the activities of these enzymes decreased over time, the excess ROS may have further exacerbated cellular damage, consistent with previous findings [46,47] (Figure 7).

## 4. Conclusions

This investigation systematically elucidates the phytotoxicological mechanisms and physiological responses of mung bean to BC, nZVI, and BC/nZVI exposures. Primary observations revealed significant (*p* < 0.05) inhibition of germination parameters, including germination rate, radicle and plumule elongation, and biomass accumulation, with minimal impact on dry matter content. Among the treatments, nZVI demonstrated the most pronounced phytotoxic effects. During vegetative growth stages, nZVI exposure caused severe growth suppression, manifested through reduced vertical growth and diminished biomass production and accompanied by progressive foliar necrosis, chlorosis, and, ultimately, plant mortality. A photosynthetic pigment analysis demonstrated substantial reductions in chlorophyll a, chlorophyll b, and carotenoid concentrations across all treatments, leading to impaired photosynthetic efficiency and compromised photoprotective mechanisms.

A comparative oxidative stress analysis revealed distinct response patterns, with nZVI and BC/nZVI exposures triggering significant upregulation in antioxidant enzymes (SOD, CAT, and POD) and abnormal iron accumulation in root tissues, exceeding physiological thresholds. The phytotoxic mechanisms involve multiple pathways: (1) direct nanoparticle adhesion to root surfaces, impeding nutrient and water uptake; (2) excessive iron accumulation disrupting ion homeostasis and nutrient transport; and (3) ROS-mediated cellular damage, resulting in chloroplast ultrastructural alterations, photosynthetic apparatus dysfunction, and, ultimately, cellular apoptosis. These findings provide comprehensive insights into the multilevel phytotoxic effects of nZVI-based materials on leguminous plants.

In summary, the modification of nZVI is crucial in affecting seedling growth, with excess iron causing significant phytotoxic effects and altering plant physiological processes. Next, we can further explain the mechanism of nZVI’s toxicological properties on plants from the perspective of metabolism and molecular omics. In addition, since the phytotoxicity of nZVI may vary with plants and their growth substances and environmental conditions, large-scale long-term nZVI experiments with different doses should be conducted in the future in order to provide more theoretical basis for the safe and green application of nZVI and its modified materials in soil remediation.

## Data Availability

The original contributions presented in this study are included in the article/Supplementary Materials. Further inquiries can be directed to the corresponding authors.

## Supplementary Materials

The following supporting information can be downloaded at: www.mdpi.com/10.3390/toxics13040250/s1, Figure S1: Germination status of mung bean seeds after 72 h exposure of different materials. (a) Control group; (b) BC treatment group; (c) nZVI treatment group; (d) BC/nZVI treatment group; Figure S2: Effect of BC, nZVI, and nZVI/BC on mung bean germination; Figure S3: Effects of BC, nZVI, and BC/nZVI on mung bean seeds after 72 h of exposure. (a) Shoot length; (b) Root length; (c) Fresh weight; (d) Dry weight; (e) and (f) are physical drawings of treated mung bean seeds.
