# Supplementary material for: Phytotoxicity of Zero-Valent Iron-Based Nanomaterials in Mung Beans: Seed Germination and Seedling Growth Experiments
Source: Toxics. 2025 Mar 27;13(4):250. doi: 10.3390/toxics13040250 (PMC12031036; doi:10.3390/toxics13040250)
Supplement: Supplementary file 1 [file toxics-13-00250-s001.zip › toxics-3509327-supplementary.pdf]

Supplementary Information

Phytotoxicity of zero-valent iron-based nanomaterials in mung beans: seed germination and seedling growth experiments

Supplementary Text S1. Preparation of materials.

Chlorella biochar (BC) was synthesized via carbothermal reduction. Before pyrolysis, oxygen was removed from the quartz tube using a vacuum pump. Chlorella powder was heated to 600 °C at 5 °C·min^{-1} in a tube furnace, held for 1 hour, and cooled to room temperature. The product was then ground through a 200-mesh sieve and stored at 60 °C in a drying box. The entire process was conducted under oxygen-free conditions.

Both nZVI and nZVI/BC were prepared using liquid-phase reduction. A solution of 5 g $FeSO_4 \cdot 7H_2O$ in 70 mL deionized water and 30 mL ethanol was sonicated for 15 minutes and transferred to a three-necked flask. Separately, 2.04 g $NaBH_4$ was dissolved in 0.1% NaOH. The $NaBH_4$ solution was added dropwise to the $FeSO_4$ solution at 1 drop/s under constant nitrogen flow to prevent oxidation. Stirring was maintained at 300 rpm, and the mixture turned black, indicating nZVI formation. After dropwise addition, nitrogen was passed for 20 minutes. The mixture was washed with ethanol 3–4 times, and solid-liquid separation was performed using a strong magnet. The resulting black solid was dried at 60 °C for 6 hours and stored.

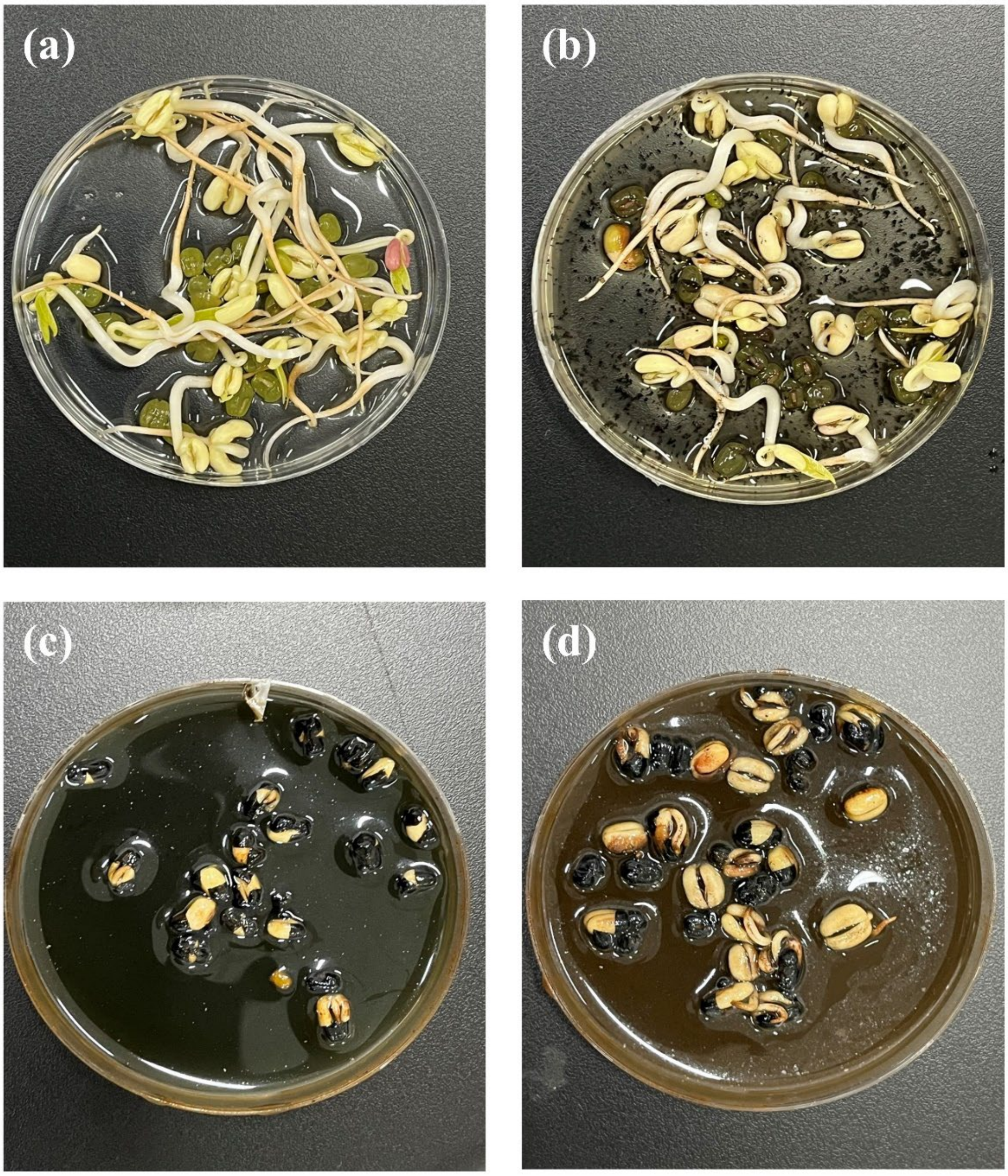

Figure S1. Germination status of mung bean seeds after 72h exposure of different materials. (a) Control group; (b) BC treatment group; (c) nZVI treatment group; (d) BC/nZVI treatment group;

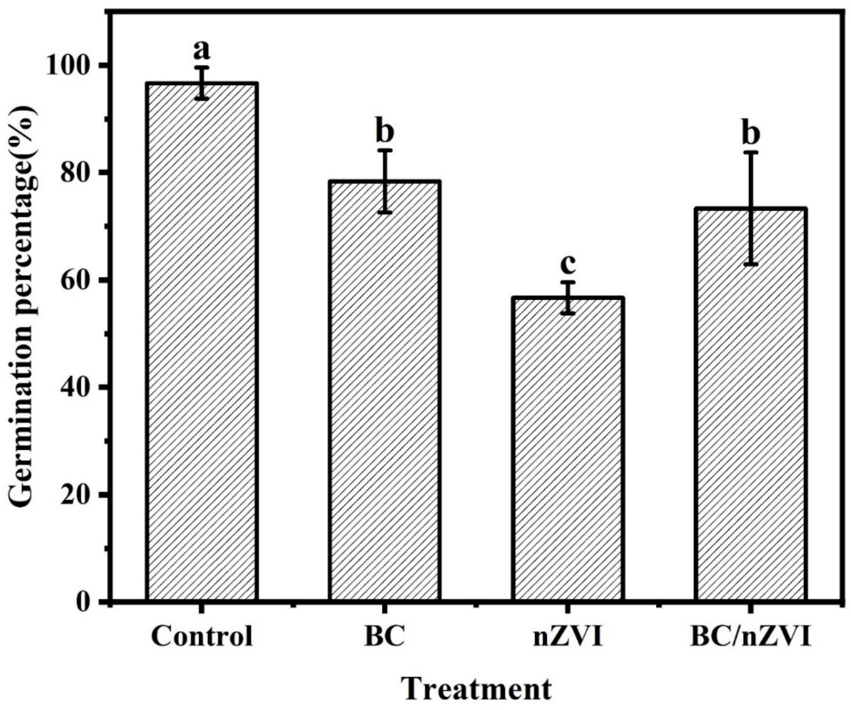

Figure S2. Effect of BC, nZVI, and nZVI/BC on mung bean germination.

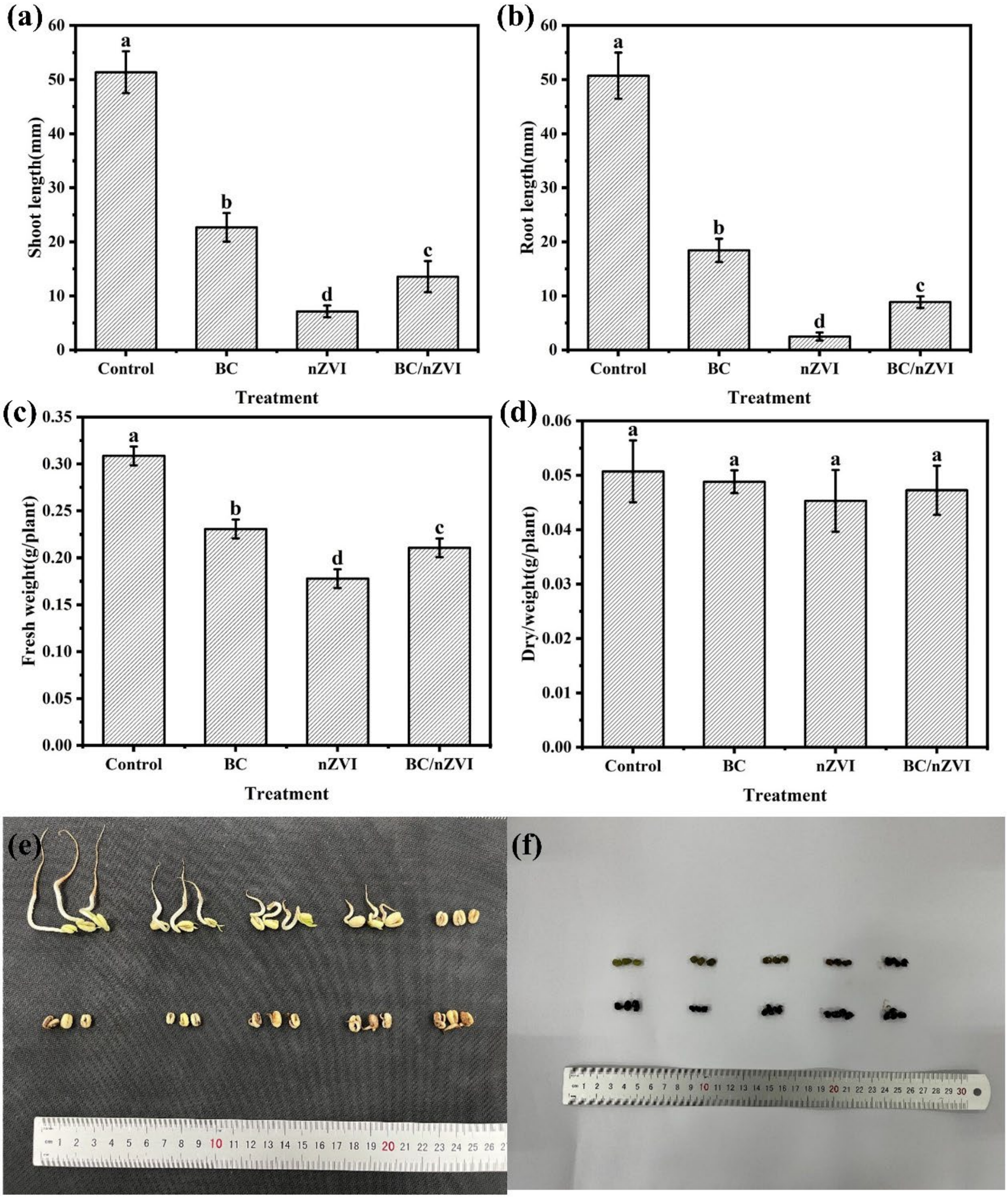

Figure S3. Effects of BC, nZVI, and BC/nZVI on mung bean seeds after 72h of exposure. (a) Shoot length; (b) Root length; (c) Fresh weight; (d) Dry weight; (e) and (f) are physical drawings of treated mung bean seeds.
